# Supplementary material for: PALI1 facilitates DNA and nucleosome binding by PRC2 and triggers an allosteric activation of catalysis
Source: Nat Commun. 2021 Jul 28;12:4592. doi: 10.1038/s41467-021-24866-3 (PMC8319299; doi:10.1038/s41467-021-24866-3)
Supplement: Supplementary file 1 — Supplementary Information [file 41467_2021_24866_MOESM1_ESM.pdf]

SUPPLEMENTARY FIGURES

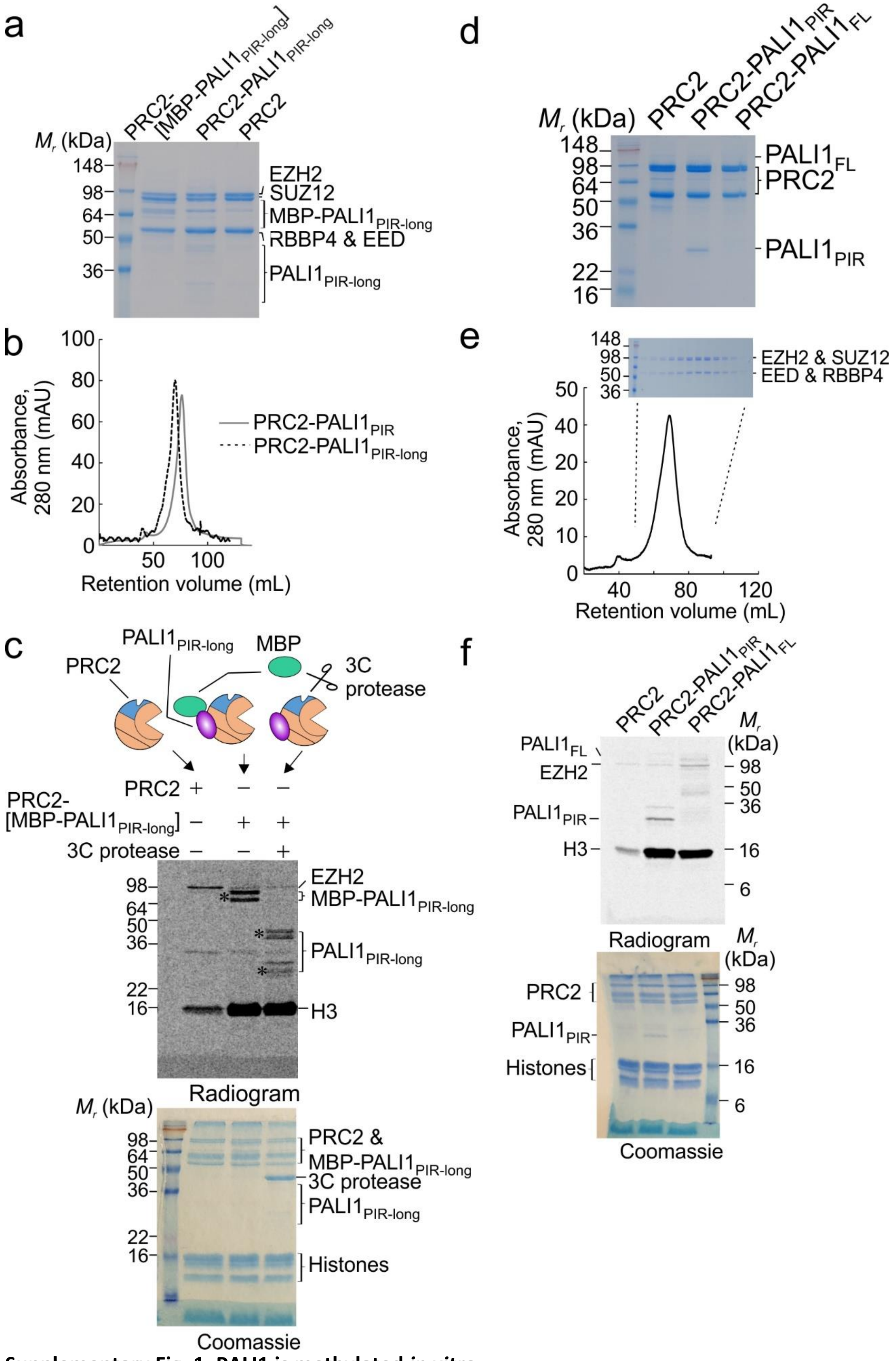

Supplementary Fig. 1. PALI1 is methylated *in vitro*.

a, Coomassie blue-stained SDS-PAGE of recombinant human PRC2-PALI1$_{PIR\text{-}long}$ complexes, as indicated. **b,** Gel filtration chromatography of the PRC2-PALI1$_{PIR}$ and PALI1$_{PIR\text{-}long}$ complexes (HiPrep 16/600 Sephacryl S-400 HR column). **c,** HTMase assay of the PRC2-[MBP-PALI1$_{PIR\text{-}long}$] complex with a mononucleosome substrate performed in the presence or absence of 3C protease to confirm that PALI1$_{PIR\text{-}long}$ is methylated. The MBP-cleaved and uncleaved PALI1$_{PIR\text{-}long}$ band indicated on the radiogram with asterisks. **d,** Coomassie blue-stained SDS-PAGE of recombinant human PRC2-PALI1$_{PIR}$ and PRC2 with a full length PALI1 (PRC2-PALI1$_{FL}$) complexes, as indicated. **e,** Gel filtration chromatography of the PRC2-PALI1$_{FL}$ (HiPrep 16/600 Sephacryl S-400 HR column). The coomassie blue-stained SDS-PAGE gel of individual fractions is presented above the chromatogram. **f,** HMTase assays were carried out using the PRC2 complexes, as indicated. HMTase assays in (**c, f**) were carried out three times with similar results, with a representative gel is presented. The numbers to the left or right hand side of the gels and radiograms in this figure represent the molecular weight marker in kDa.

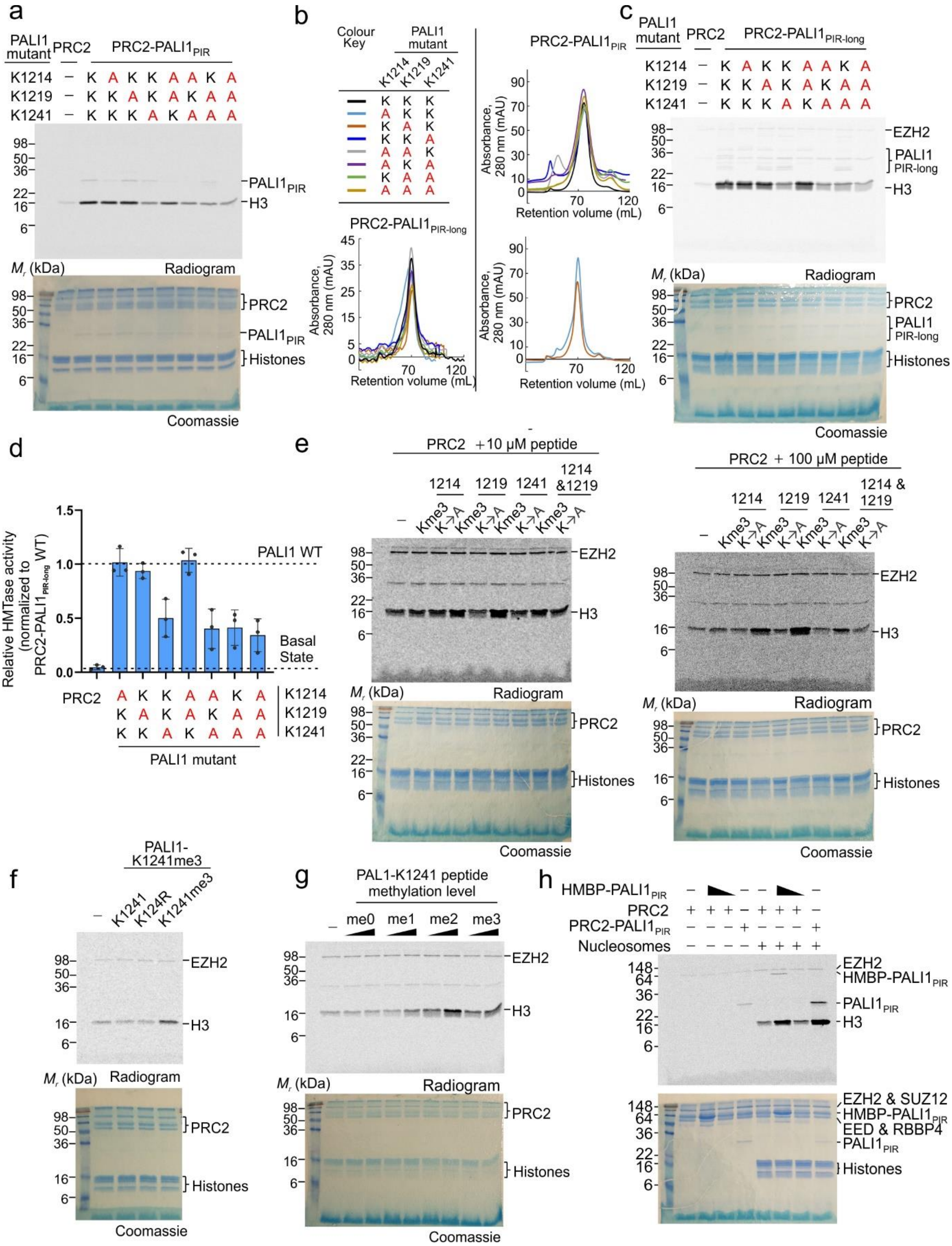

Supplementary Fig. 2. PALI1-K1241me2/3 is required and sufficient to stimulate the HMTase activity of PRC2.

a, A representative uncropped radiogram and the corresponding uncropped Coomassie blue-stained SDS-PAGE, as shown in Fig. 2a. **b,** Gel filtration chromatography (HiPrep 16/600 Sephacryl S-400 HR column) of the PRC2-PALI1$_{PIR-long}$ (left) and PRC2-PALI1$_{PIR}$ (right) wild-type and mutants, as indicated. **c,** HMTase assays were carried out using wild-type or mutant recombinant PRC2-PALI1$_{PIR-long}$ complexes, as indicated. **d,** The bar plot represents mean HMTase activities from three independent

replicates (n=3) with the error bars represent standard deviation, quantified using densitometry and normalized to the activity of the wild-type PRC2-PALI1$_{PIR\text{-}long}$. Dash lines indicate the activity of the wild-type PRC2-PALI1$_{PIR\text{-}long}$ (upper line) and the core PRC2 (bottom line) complexes. **e,** A representative full radiogram and the corresponding uncropped Coomassie blue-stained SDS-PAGE as shown in Fig. 2b for 10 µM (left) and 100 µM (right) peptide concentration. **f,** A representative full radiogram and the corresponding uncropped Coomassie blue-stained SDS-PAGE as shown in Fig. 2c . **g,** A representative full radiogram and the corresponding uncropped Coomassie blue-stained SDS-PAGE as shown in Fig. 2d. **h,** HMTase assays were carried out in the presence or absence of mononucleosome substrates using PRC2-PALI1$_{PIR}$ or PRC2 supplemented with different concentrations of N-terminus 6xHis-MBP tagged PALI1$_{PIR}$ protein, which was purified from insect cells without the co-expression of other PRC2 subunits. The assays were repeated three times with similar results, with a representative gel is presented. The numbers on the left-hand side of the gels and radiograms in this figure represent the molecular weight marker in kDa.

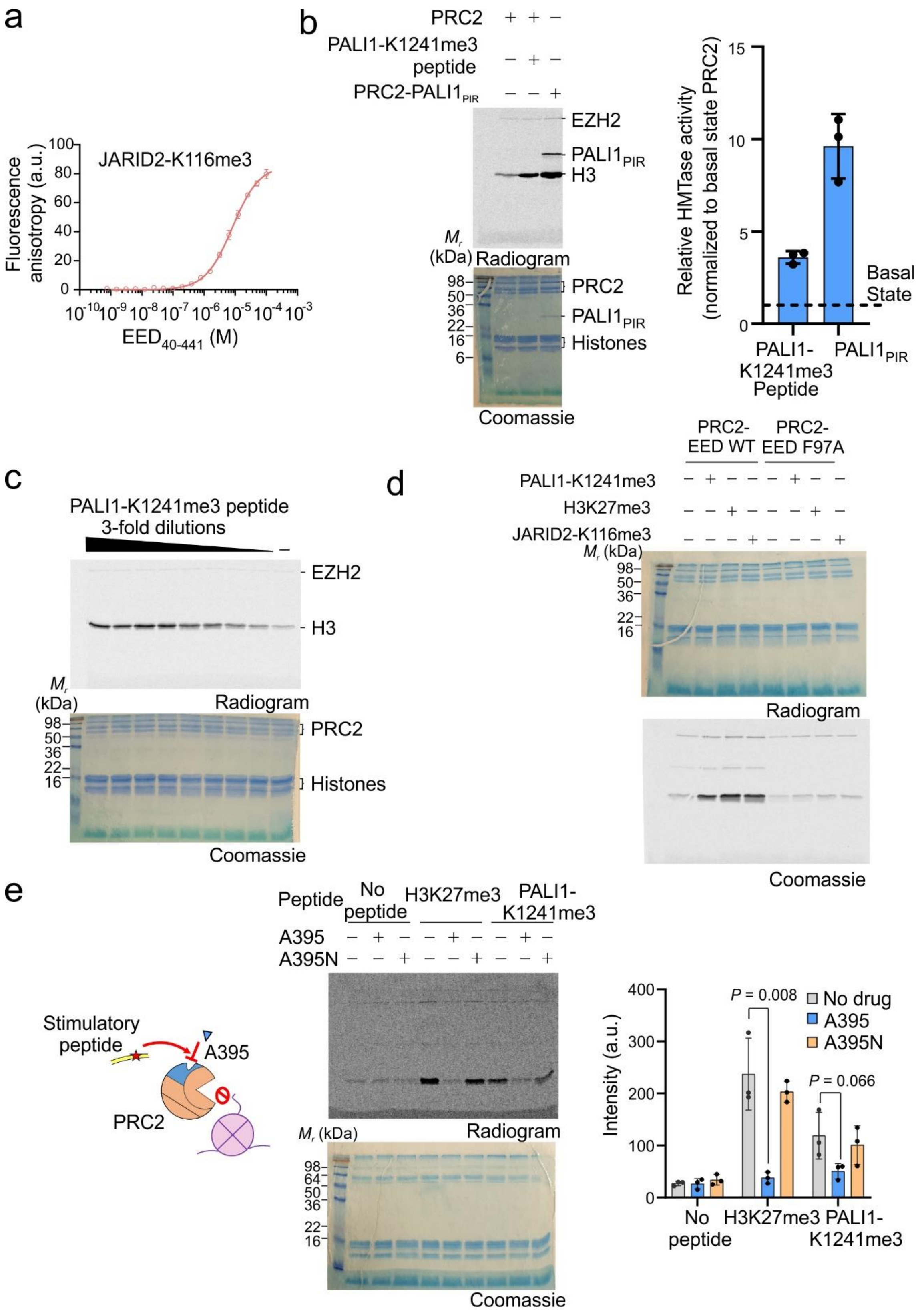

Supplementary Fig. 3. PALI1-K1241me2/3 binds to the aromatic cage of the regulatory subunit EED to stimulate PRC2.

a, Fluorescence anisotropy performed to quantify the binding affinity of EED for the 5-FAM labelled JARID2-K116me3 peptide. Each data points in the plot indicate the mean of the normalised anisotropy values and the error bars represent standard deviation over three independent

experiments carried out in different days. Dissociation constants (K_d) and 95% confidence bounds on the coefficient are indicated in Fig. 3a. **b,** HMTase assays performed using PRC2 in the absence or presence of stimulatory peptide or PRC2-PALI1$_{PIR}$, as indicated. The bar plot represents the mean of the relative HMTase activities, normalized to the HMTase activity of PRC2 in its basal state (dashed line). Error bars represent standard deviation from three independent replicates. **c,** A representative full radiogram and the corresponding uncropped Coomassie blue-stained SDS-PAGE as shown in Fig. 3b**. d,** A representative uncropped radiogram and the corresponding uncropped Coomassie blue-stained SDS-PAGE as shown in Fig. 3d**. e,** HMTase assay of 200 nM PRC2 using 2 μM mononucleosomes, in the presence or absence of 50 μM stimulatory peptides, as indicated, and in the presence or absence of 0.8 μM allosteric inhibitor A395 or the negative control compound A395N. The bar plot represents the means of quantification using densitometry from three independent replicates with the error bars represent standard deviation. P-values were determined using unpaired two-tailed Student's t-test.

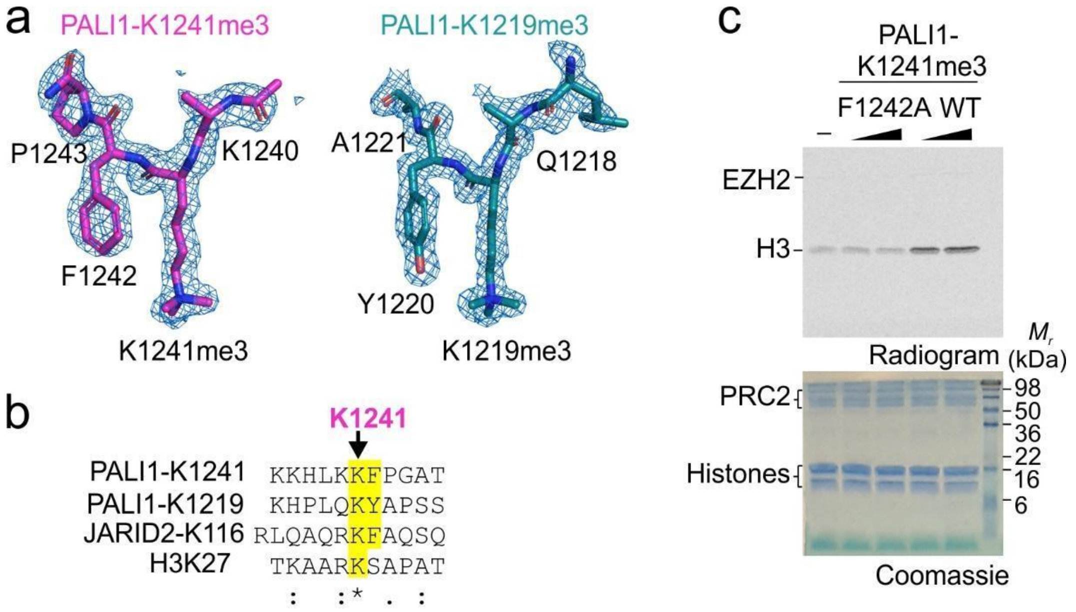

Supplementary Fig. 4. Structural basis for a convergent evolution between PALI1 to JARID2.

a, Fo-Fc omit electron density maps for PALI1 peptides bound to EED, contoured at 3.0 σ. Omit map was calculated using Polder Maps (Liebschner et al. 2017) in PHENIX (Liebschner et al. 2019) visualized using PyMOL (The PyMOL Molecular Graphics System, Version 2.0 Schrödinger, LLC.). **b,** The sequences of the tri-methyl-lysine peptides used for the crystallization of EED, including the PALI1 peptides (this study), the JARID-K116me3 peptide (Sanulli et al. 2015) and the H3K27me3 peptide (Margueron et al. 2009), aligned according to the methylated lysine residues. The methyl-lysine and the adjacent aromatic amino acids at the +1 position are highlighted in each of the peptides, when applicable. **c,** A representative full radiogram and the corresponding uncropped Coomassie blue-stained SDS-PAGE as shown in Fig. 4d**.**

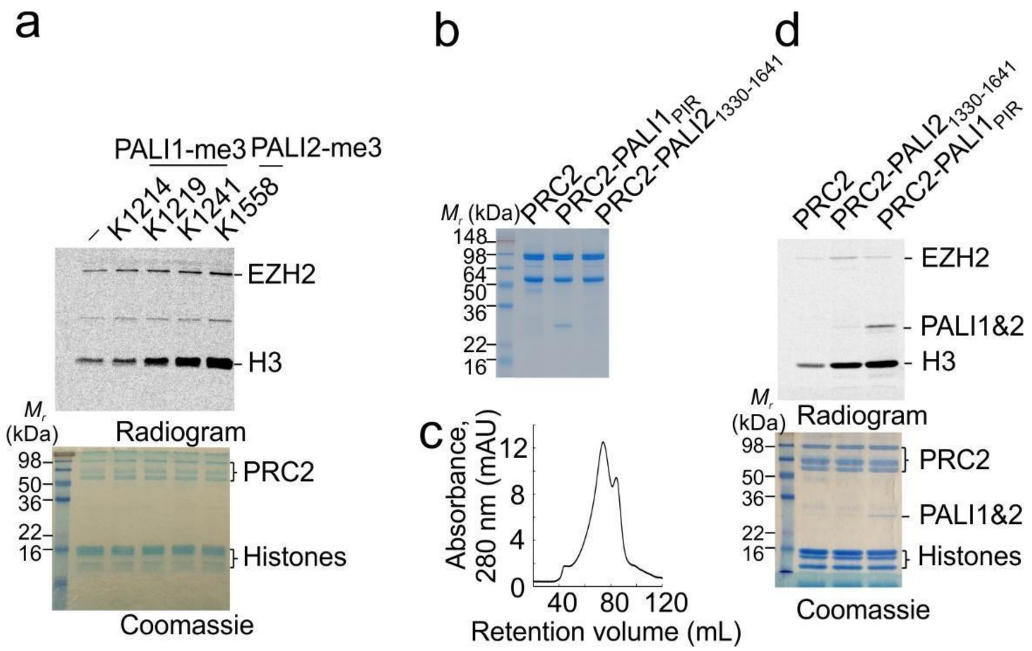

Supplementary Fig. 5. PALI2-K1558me3 mimics PALI1-K1241me3 in the allosteric activation of PRC2 *in vitro*.

a, A representative full radiogram and the corresponding uncropped Coomassie blue-stained SDS-PAGE as shown in Fig. 5c**. b,** Coomassie blue-stained SDS-PAGE of recombinant PRC2 complexes, as indicated. **c,** Gel filtration chromatography of the PRC2-PALI2$_{1330\text{-}1641}$ complex (HiPrep 16/600 Sephacryl S-400 HR column). **d,** A representative full radiogram and the corresponding uncropped Coomassie blue-stained SDS-PAGE as shown in Fig. 5d.

a

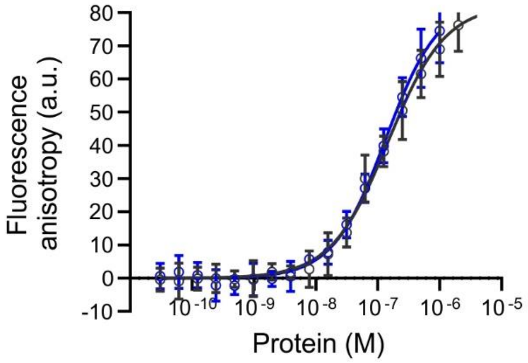

Key	Protein	Probe	K_d (nM)	Hill
	PRC2	G-tract RNA	147 ± 22	0.94 ± 0.09
	PRC2-PALI1$_{PIR}$	G-tract RNA	134 ± 25	1.02± 0.11

b

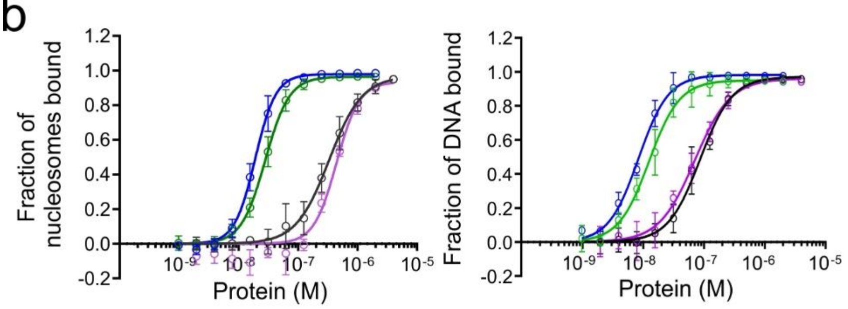

Key	Protein	Nucleosome binding		DNA binding	
		K_d (nM)	Hill	K_d (nM)	Hill
	PRC2	333 ± 30	1.56 ± 0.17	86.2 ± 5.5	1.65 ± 0.14
	PRC2 + 100 µM PALI1-K1241me3 peptide	444 ± 37	2.27 ± 0.36	70.6 ± 5.6	1.48 ± 0.14
	PRC2-PALI1$_{PIR}$	19.0 ± 0.6	2.49 ± 0.18	8.35 ± 0.34	1.76 ± 0.11
	PRC2-PALI1$_{PIR}$ K1241A	28.1 ± 1.0	2.15 ± 0.14	12.5 ± 0.8	1.73 ± 0.17

c

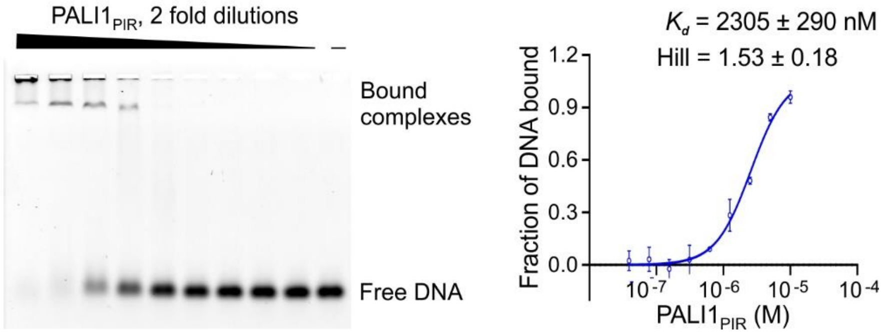

Supplementary Fig. 6. PALI1 is a DNA binding subunit of PRC2.

a, Fluorescence anisotropy was performed to quantify the affinity of PRC2 complexes for G4 24 RNA (UUAGGG)$_4$. Data represent the mean of three independent experiments that were carried out on different days. Error bars represent standard deviation. Standard errors of dissociation constants (K_d)

and Hill coefficients are indicated in the table. **b,** Quantification of EMSA from Fig. 4b. The affinities of the indicated PRC2 complexes to mononucleosomes and free-DNA, from data shown in Fig. 4b, were quantified using densitometry from the free-nucleosome and free-DNA bands. Data represent the mean of three independent experiments and the error bars represent standard deviations. Standard errors of dissociation constants (K_d) and Hill coefficients are indicated in the table. **c,** EMSA used to quantify the affinity of PALI1$_{PIR}$ (MBP tag was removed from the N-terminal) to a fluorescein-labelled CpG46 DNA. Data represent the mean of three replicates and error bars represent standard deviation. Dissociation constants (K_d) and Hill coefficients are indicated, including their standard error.

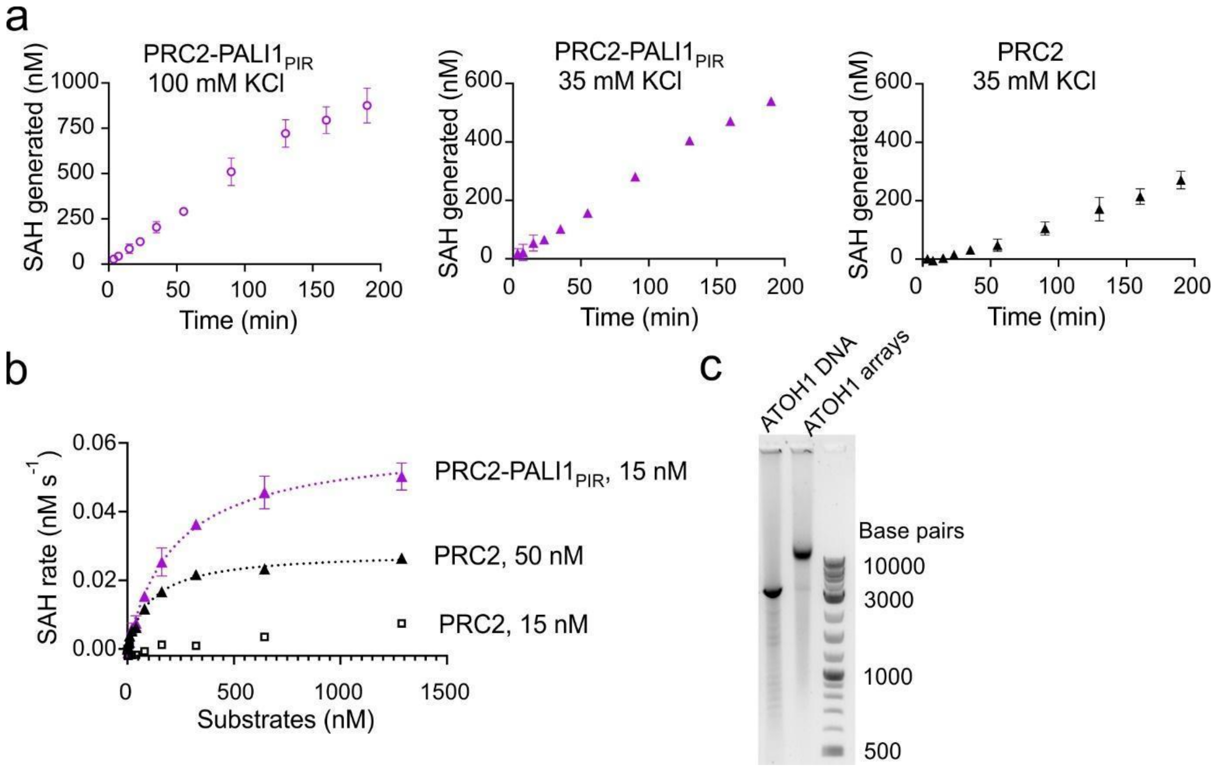

Supplementary Fig. 7. Supporting material for quantitative histone methyltransferase assays.

a, Progress curves of PRC2 complexes on a nucleosomal array substrate, with the same color keys were used as in Fig. 7c. Each data point in the plot indicate the mean value of SAH generated and the error bar represents the standard deviation from three independet replicates carried out on three different days. **b,** Substrate titration experiment, with the enzymes and their concentrations are indicated. Data is as in Fig. 7c, except for the data series of PRC2 at 15 nM enzyme concentration that was added here for a direct comparison with PRC2-PALI1$_{PIR}$ that was assayed at 15 nM enzyme concentration. The monovalent ion in all assays is 35 mM KCl. Data represents the mean of three independent replicates (n=3) that were carried out on different days and error bars represent standard deviation. **c,** The nucleosomal arrays used for kinetic assays in Fig. 7c and here, derived from a 3.6 kb long DNA bearing the sequence of the ATOH1 gene, were analysed on a 0.8 % agarose TBE gel and visualized by Sybr Safe.

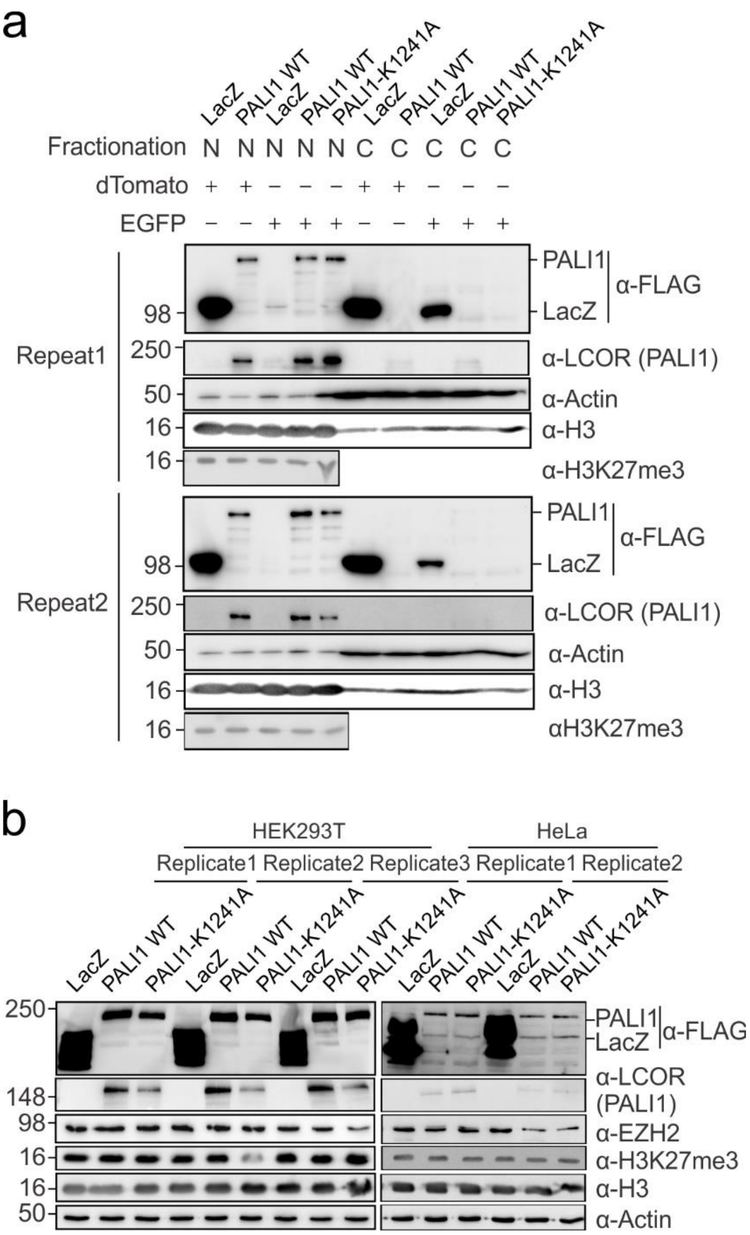

Supplementary Fig. 8. Western blots of PALI1 overexpressing in cells.

a, Nuclear and cytoplasmic fractions of K562 cells overexpressing different proteins were isolated and examined by western blotting with antibodies as indicated. Results are from two independent replicates are shown. Subcellular fractions are abbreviated as N for nuclear fraction and C for cytoplasmic fraction. **b,** Western blots of HEK293T and HeLa cells overexpressing proteins as indicated performed using antibodies as indicated. The H3 and EZH2 blots originated from the same membrane that was split after the transfer and the Actin and H3K27me3 blots are originated from the same membrane that was split after the transfer. In (**a**) and (**b**), results from two or three independent replicates are shown. Different blots were subjected to SDS-PAGE and bloting seperately unless otherwise indicated, with the uncropped source data are provided as a Source Data file.

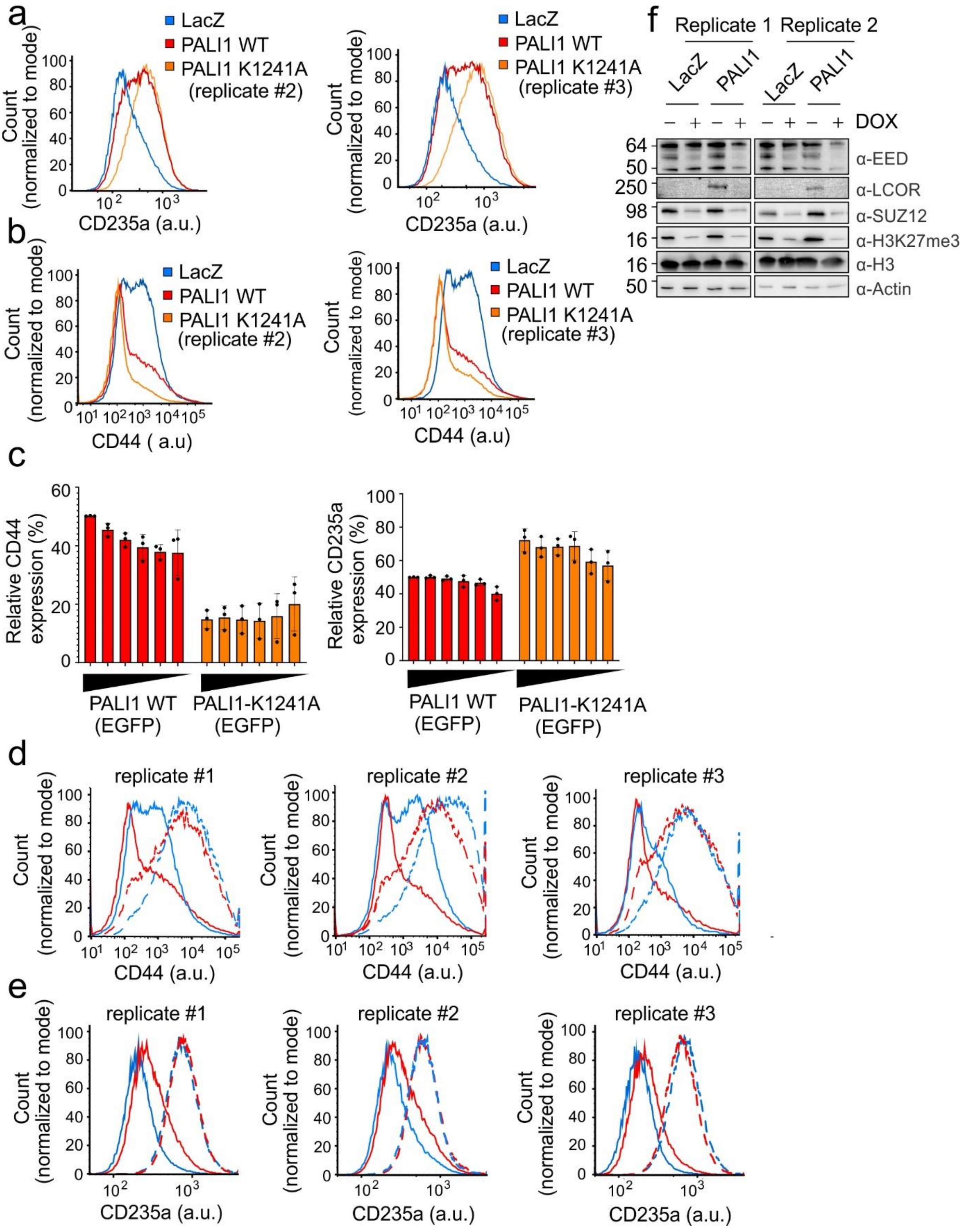

Supplementary Fig. 9. PALI1 stability and changes in the expression of CD markers are dependent on PRC2, and independent of the expression level of PALI1

a, b, The two replicates of the histograms representing the distribution of cells based on the expression of the erythroid marker CD235a (a) or CD44 (b) as detected by flow cytometric analysis of K562 cells overexpressing different proteins, as indicated (Blue: LacZ, red: PALI1 WT, orange: PALI1

K1241A). Results are from three independent experiments that were carried out on different days, starting each time from lentiviral transduction, with the other replicate presented in a body figure (Fig. 8d). **c,** Bar charts representing the percentage of cells exhibiting a higher CD marker expression level with respect to cells expressing a high level of ectopically expressed PALI1 WT (in red) or PALI1-K1241A (in orange), carried out to control against the possibility that results in Fig. 8d are driven by small variations in the expression level of the different constructs. K562 cells were transduced with lentiviruses for the expression of PALI1 WT or PALI1-K1241A using an EGFP polycistronic vector, as described for experiments in Fig. 8d. The only difference is that here, cells were gated 7 days post transduction into separate plates according to the expression level of the EGFP, which served as a proxy for the expression level of the ectopically expressed mRNA. During this process, six new samples were created from each culture, representing the top six deciles of cells based on their EGFP expression level. Each of these six groups of cells were then cultured separately for an additional 7 days, until day 14 (from the transduction) when CD marker quantification was carried out using flow cytometry, as described for the experiments carried out in Fig 8d. Means (i.e. y-axes) represent the percentage of cells from each decile that their CD marker of interest is expressed higher than in the median of the 10th decile group. Each group of same-coloured bars represents the top 6 deciles, ordered from the 10th decile in the left (i.e. highest expression level of PALI1: 90%-100% percentile) to the 5th decile in the right (i.e. less than the median expression level of PALI1: 40%-50% percentile). Results are from three independent replicates (n=3) that were carried out on different days and the error bars represent standard deviation. **d, e,** Histograms representing the distribution of cells based on the expression of the erythroid marker CD44 (d) and CD235a (e) as detected by flow cytometric analysis of K562 cells overexpressing LacZ (blue line) or PALI1 (red line) with (dashed line) or without (continuous line) doxycycline-inducible CRISPR/Cas9 knockout of EED. **f,** Immunoblotting was carried out on K562 cells using antibodies as indicated in the presence or absence of doxycycline-inducible CRISPR/Cas9 knockout of EED. The EED and H3 blots originated from the same membrane that was split after the transfer. The Actin and H3K27me3 blots originated from the same blot that was split after the transfer. The LCOR and SUZ12 blots originated from the same membrane that was split after the transfer. Source data are provided as a Source Data file and gating strategies are shown in Supplementary Fig. 11.

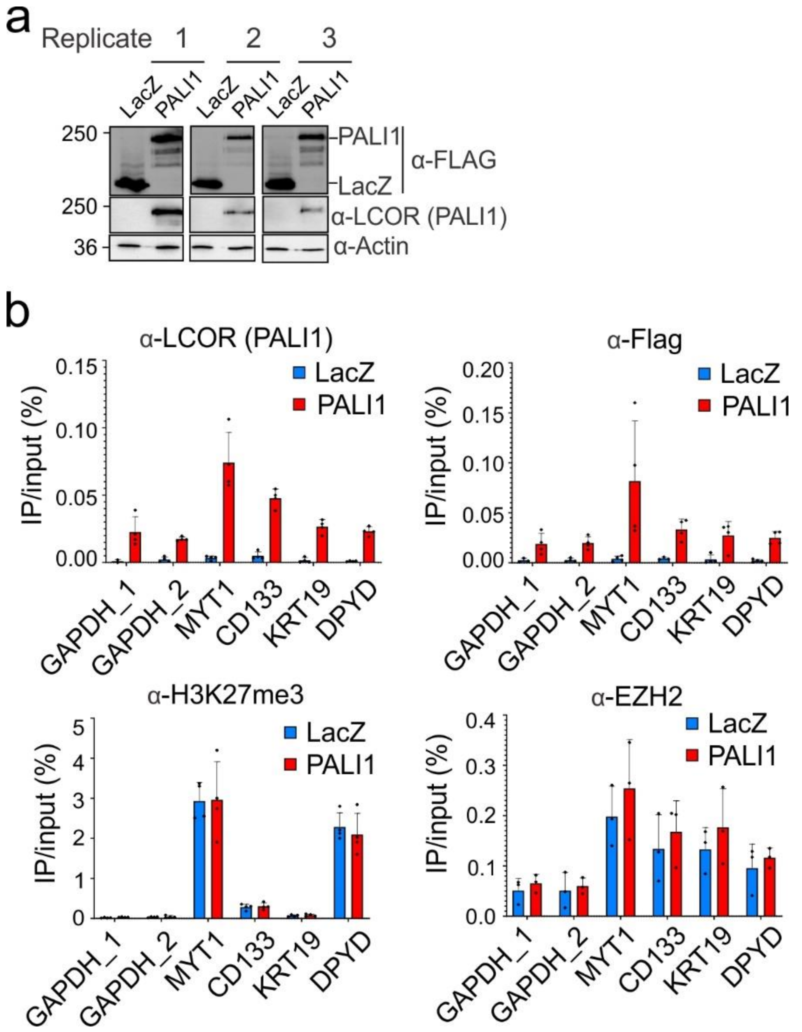

Supplementary Fig. 10. Overexpressed PALI1 binds to chromatin in HEK293T cells.

a. Immunoblotting of the overexpressed PALI1 and the negative control LacZ in HEK293T cells from three independent replicates. Each blot originated from a separate membrane with the uncropped source data are provided as a Source Data file. **b,** ChIP-qPCR was carried out using antibodies as indicated on selected genes, as indicated. Means in the bar plots represent the IP over the input and the error bars represent the standard deviation from three independent replicates that were carried out on different days.

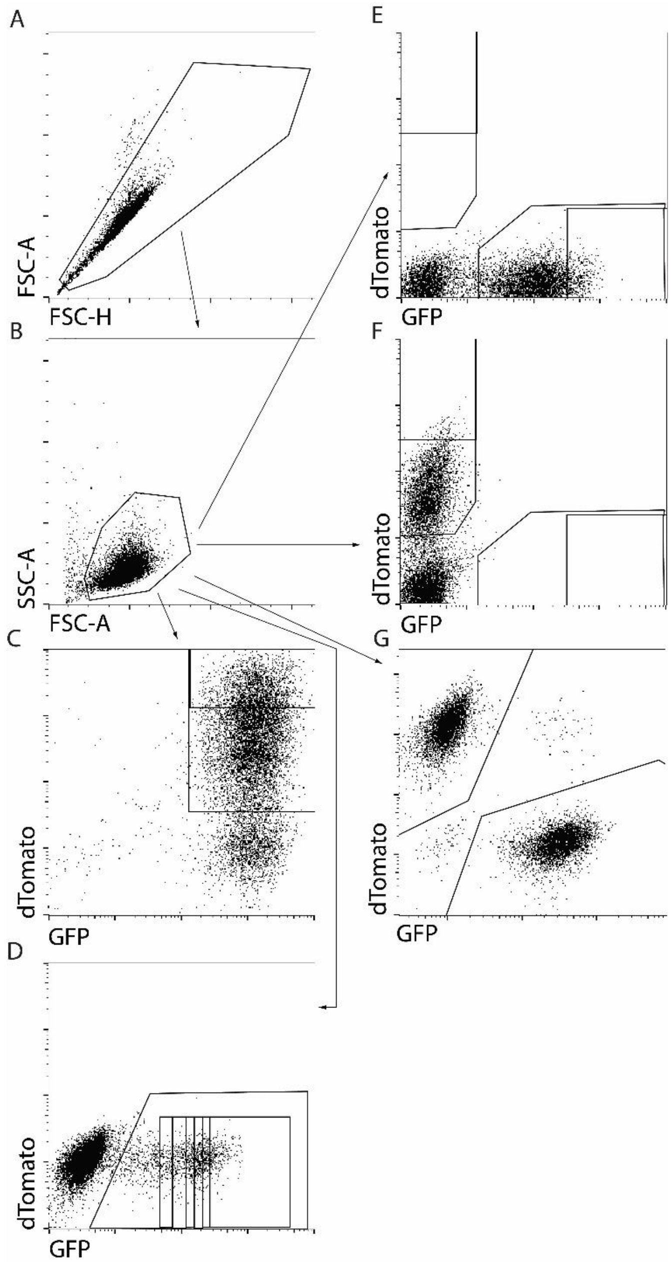

Supplementary Fig. 11. Gating Strategies used for cell sorting.

Representative dot plots showing the gating strategies used for flow cytometry. In all experiments, a gate was first set up to include single cells (**a**), followed by another gate to include intact K562 cells (**b**). (**c**) Gating strategy for sorting GFP (EED gRNA) and dTomato (LacZ or PALI1) positive cells, used in Supplementary Fig. 9d,e,f. (**d**) Gating strategy for sorting GFP (PALI1 wt or PALI1 K1241A) cells from the last 6 deciles of expression, used in Supplementary Fig. 9c. Gating strategy for sorting GFP (**e**) or dTomato (**f**) (PALI1 wt or PALI1 K1241A), used in Fig. 8b,c,d. (**c-f**) The outer gates contain the GFP or dTomato positive cells, and the inner gates were used for sorting. (**g**) Gating strategy to identify GFP or dTomato (LacZ, PALI1 wt, or PALI1 K1241A) positive cells, used in all analytical flow cytometry experiments.

SUPPLEMENTARY MATERIAL

Supplementary Data 1. The summary of the PRC2 methylome *in vivo* and *in vitro*. Residues with a position probability of less than 0.95 were indicated with red text and probability scores shown in parentheses. Residues from peptides that were ambiguous between EZH1 and EZH2 are indicated by an asterisk.

Supplementary Table 1. Primers and sequences used in this study.

Cloning Primers

PALI1_F2_pFB1.HMBP.PrS GGAAGTTCTGTTCCAGGGGCCCGGGCAGCGAATGATCCAACAATTTG

PALI1_R1557_pFB1.HMBP.PrS
ATGCCTCGAGACTGCAGGCTCTAGATTATCACTTTGCATCCAGCCGCCTCCG

PALI1_R310_inter GACTTTCTACTAAAGCTGAACTCTGCATATGGTCTTTACCATCCTCACA

PALI1_F312_inter CATATGTGAGGATGGTAAAGACCATATGCAGAGTTCAGCTTTAGTAGAA

PALI1_F1058_pFB1.HMBP.PrS GGAAGTTCTGTTCCAGGGGCCCGGGACTTCAGAAAAGGAAGCTGC

PALI1_R1329_pFB1.HMBP.PrS
ATGCCTCGAGACTGCAGGCTCTAGATTATCAATTCTTGATTAAACGTTGCTG

PALI1_R1250_pFB1.HMBP.PrS
ATGCCTCGAGACTGCAGGCTCTAGATTATCAATTCTTAGCAGGGGTAGCTCC

EED_F40_pGEX-MHL ttgtatttccagggcGACGCTGTCAGTATAGAAAGTG

EED_F76_pGEX-MHL ttgtatttccagggcAAGAAATGCAAATATTCTTTCAAATG

EED_R441_pGEX-MHL caagcttcgtcatcaTCGAAGTCGATCCCAGCGC

PALI1_F1058_pMAL-mhl TTGTATTTCCAGGGCACTTCAGAAAAGGAAGCTGCAC

PALI1_R1250_pMAL-mhl CAAGCTTCGTCATCAATTCTTAGCAGGGGTAGCTCC

PALI1_F1_pHIV-EGFP/pHIV-dTomato
AACTATTCTAGAGTACCCACCATGGACTACAAAGACGATGACGACAAGATGCAGCGAATGATCCAAC
AA

PALI1_R1557_pHIV-EGFP/pHIV-dTomato
AGGGGCGGATCCTAGCCCCTATTATACCTTTCTCTTCTTTTTTGGCTTTGCATCCAGCCGCCTCCG

LacZ_F_pHIV-EGFP/ pHIV-dTomato
AACTATTCTAGAGTACCCACCATGGACTACAAAGACGATGACGACAAGGTCGTTTTACAACGTCGTG
AC

LacZ_R_pHIV-EGFP/pHIV-dTomato
AGGGGCGGATCCTAGCCCCTATTATACCTTTCTCTTCTTTTTTGGTTTTTGACACCAGACCAACTG

Primers used to generate point mutations

PALI1_K1241A_F TTGAAGGCTTTTCCTGGAGCTACCCCT

PALI1_K1241A_R AGGAAAAGCCTTCAAGTGCTTTTTCAA

PALI1_K1214A_F CCTGTCGCTCATCCTCTTCAGAAATAC

PALI1_K1214A_R AGGATGAGCGACAGGGGGAACGTCTCC

PALI1_K1219A_F CTTCAGGCTTACGCTCCTTCCAGCCTA

PALI1_K1219A_R AGCGTAAGCCTGAAGAGGATGCTTGAC

PALI1_K1214A/K1219A_R AGCGTAAGCCTGAAGAGGATGAGCGAC

Primers used to amplify 147 and 182-base-pair DNA for the reconstitutation of mononuclesomes

147_fw CTGGAGAATCCCGGTGCCG

147_rev ACAGGATGTATATATCTGACACG

182_fw ACCTCGCGAATGCATCTAGAT

182_rev AGGGCGCCGATATCGGAT

Primers used to amplify ATOH1 gene from purified K562 genomic DNA

ATOH1_gb_fw GTCGACTCTAGAGGATCCCCGCAGAGCCCA

ATOH1_gb_rev CGAATTCGAGCTCGGTACCCGCGGAGTTTCCTAAAAGACGCC

Primers used to amplify ATOH1 from pUC18 plasmid

ATOH1_fw GCAGAGCCCAAACATTCACACA

ATPH1_rev GCGGAGTTTCCTAAAAGACGCC

Primers used for ChIP-qPCR

GAPDH_1_fw GACCTCTTTTCCCACTTTTTC

GAPDH_1_rev TTTCATTCCATCCAGCCTG

GAPDH_2_fw GCACACTGTCTCTCTCCCTAG

GAPDH_2_rev ATTAGGGCAGACAATCCCGGC

MYT-1_fw AGGCACCTTCTGTTGGCCGA

MYT-1_rev AGGCAGCTGCCTCCCGTACA

CD133_fw CCCAGTGGATGGAAAGAAGA

CD133_rev ACTGGGGGTGTACAGTGAGG

KRT19_fw GTCGCGGATCTTCACCTCTA

KRT19_rev TTTGTGTCCTCGTCCTCCTC

DPYD_fw TCTAGCTCATGAATCACGGGT

DPYD_rev ACAGCACCTTACTTTTCCCTCAA

Primers for cloning EED gRNA

EED gRNA H1 F TCCCAAGAGAATGATCCATACCAC

EED gRNA H1 R AAACGTGGTATGGATCATTCTCTT

147-base-pair DNA sequence

CTGGAGAATCCCGGTGCCGAGGCCGCTCAATTGGTCGTAGACAGCTCTAGCACCGCTTAAACGCACGTACGC
GCTGTCCCCCGCGTTTTAACCGCCAAGGGGATTACTCCCTAGTCTCCAGGCACGTGTCAGATATATACATCCTG
T

DNA probes used for DNA binding assays using fluorescence anisotropy

CpG46 (3'-fluorescein-labelled)
5'GGCGCCCTGCCCCGCCTCGCTCTGGCAGAGTGGGGAGCCAGCCGGCGCTAGCCGGCTGGCTCCCCACTCTG
CCAGAGCGAGGCGGGGCAGGGCGCC

CpG46 mt (3'-fluorescein-labelled)
5'AATATTTCATTTTATTCTATCTCAATGAGACAAAAGATTGATTAATGCTAATTAATCAATCTTTTGTCTCATTG
AGATAGAATAAAATGAAATATT

182-base-pair DNA sequence

ACCTCGCGAATGCATCTAGATGATATCGAGAATCCCGGTGCCGAGGCCGCTCAATTGGTCGTAGACAGCTCTA
GCACCGCTTAAACGCACGTACGCGCTGTCCCCCGCGTTTTAACCGCCAAGGGGATTACTCCCTAGTCTCCAGG
CACGTGTCAGATATATACATCCGATATCGGCGCCCT

PALI1 full-length sequence

ATGCAGCGAATGATCCAACAATTTGCTGCTGAATATACCTCAAAAAATAGCTCTACTCAGGACCCCAGCCAGC
CCAATAGCACAAAGAACCAAAGCCTGCCGAAAGCATCTCCAGTCACCACCTCTCCCACGGCTGCAACTACTCA
GAACCCTGTGCTCAGCAAACTTCTCATGGCTGACCAAGACTCACCTCTGGACCTTACTGTCAGAAAGTCTCAGT
CAGAACCTAGCGAACAAGACGGTGTACTTGATCTGTCCACTAAGAAAAGTCCATGTGCTGGCAGCACTTCCCT
GAGCCACTCTCCAGGCTGCTCCAGTACTCAAGGGAACGGTGAGAACTCAACAGAGGCAAAAGCAGTAGATTC
TAACAATCAGTCGAAGTCCCCACTGGAGAAATTTATGGTCAAACTGTGTACTCATCATCAAAAGCAATTCATTC
GTGTTCTGAACGACCTGTACACTGAATCTCAACCAGGCACTGAGGACCTGCAGCCTTCTGATTCGGGAGCAAT
GGATGTATCCACTTGCAATGCTGGCTGTGCCCAGCTCAGCACCAAACATAAGGAAAAAGATGCTCTGTGTCTC
GATATGAAGTCTTCTGCTTCTGTAGATTTGTTCGTAGACTCGTCAGACTCTCACAGCCCTCTACACTTGACGGA
ACAGACCCCGAAGAAGCCTCCTCCTGAGATAAACCCTGTAGATGGAAGAGAGAATGCCTTGACTGTTGTCCA
GAAAGATTCCTCTGAACTTCCAACCACTAAATCGAATTCTATTAATAGCAGTTCAGTGGATAGTTTCACTCCGG
GATACCTCACTGCATCTAATTGTTCCTCAGTGAACTTCCACCACATCCCTAAAATCTTGGAGGGGCAGACCACT
GGACAAGAGCAAGACACAAATGTGAACATATGTGAGGATGGTAAAGACCATATGCAGAGTTCAGCTTTAGTA
GAAAGTCTAATTACAGTAAAAATGGCAGCTGAGAATAGTGAGGAAGGCAATACCTGTATTATTCCTCAAAGA
AATTTGTTCAAAGCTTTATCAGAAGAGGCTTGGAACTCAGGGTTTATGGGGAACTCATCTAGAACTGCTGACA
AAGAGAATACTTTACAGTGTCCAAAAACACCTTTGCGCCAGGATTTAGAGGCAAATGAACAAGATGCAAGGC
CAAAGCAAGAGAACCATCTTCACTCTCTGGGAAGAAATAAGGTGGGTTACCATTTACATCCCAGTGATAAGGG
CCAGTTTGATCATTCCAAAGATGGTTGGTTAGGCCCCGGCCCTATGCCAGCTGTACACAAAGCGGCAAATGGA
CACTCAAGAACCAAGATGATATCAACCTCCATCAAGACAGCTCGGAAAAGTAAAAGGGCATCAGGGCTGAGG
ATAAATGATTATGATAACCAGTGTGATGTTGTTTATATCAGTCAACCAATAACAGAATGCCACTTTGAGAATCA
AAAATCAATATTATCTTCTCGGAAAACAGCCAGAAAGAGTACTCGAGGATACTTTTTCAATGGTGACTGTTGT
GAGCTGCCAACTGTTCGTACACTGGCCAGAAATTTACACTCCCAGGAAAAAGCAAGCTGCTCAGCATTGGCAT
CAGAGGCAGTTTTCACTCCTAAGCAGACCCTTACAATTCCAGCCCCTAGACATACAGTAGATGTGCAGCTTCCC
AGAGAAGACAACCCTGAAGAACCTAGCAAGGAAATCACCTCTCACGAGGAAGGAGGTGGAGACGTTTCACCT
CGAAAAGAACCTCAAGAGCCTGAGGTTTGCCCCACAAAGATTAAGCCGAACCTGAGCAGCTCCCCTAGGTCA
GAGGAAACGACAGCCTCCAGCCTGGTGTGGCCTCTCCCTGCTCACCTTCCTGAAGAGGACCTGCCAGAAGGT
GGCTCCACAGTCTCAGCTCCCACAGCAAGTGGGATGTCTTCTCCTGAACACAACCAACCACCAGTTGCACTGTT
GGATACGGAGGAGATGAGTGTACCCCAGGACTGTCACCTCCTTCCCTCCACTGAAAGCTTTTCCGGGGGAGTC
AGTGAAGATGTCATTTCTAGGCCTCATTCTCCTCCTGAAATAGTCAGTAGAGAAGAAAGTCCTCAGTGCTCAG
AAAATCAGAGTTCCCCAATGGGCTTGGAGCCCCCCATGAGTCTGGGAAAGGCTGAGGACAACCAAAGCATCA
GTGCTGAGGTTGAGTCTGGAGACACCCAGGAGCTAAATGTCGACCCACTCTTGAAGGAAAGCAGCACTTTTA

CTGATGAAAACCCCAGTGAAACTGAGGAAAGTGAGGCAGCAGGTGGTATAGGAAAATTAGAGGGAGAGGA
CGGTGATGTAAAATGCCTGTCAGAAAAAGACACGTATGATACAAGCATTGACTCACTCGAAGAGAATTTGGA
CAAGAAGAAAAAAGGTAAAAAATTCCCTGAGGCCTCTGATAGGTGCCTAAGAAGTCAACTTTCGGATTCTTCC
TCTGCTGACAGATGCCTAAGAAATCAGAGTTCAGATTCTTCCTCAGCTTGTCTTGAAATCAAAGTTCCTAAAAA
TCCTAGTGCAAAACGTTCAAAAAAAGAAGGGCACCCTGGTGGGACAACACCTAAGGGCCTTCTACCTGACAG
TTTCCACACGGAAACTCTGGAGGACACAGAAAAGCCAAGTGTCAATGAACGCCCCTCTGAGAAAGATGCTGA
GCAGGAGGGCGAAGGCGGGGGGATCATCACCAGGCAGACTTTGAAAAACATGCTGGACAAAGAAGTCAAG
GAGTTACGAGGAGAGATTTTCCCCAGCAGGGACCCCATAACCACAGCTGGACAGCCACTGCCTGGAGAGAGA
TTGGAAATCTATGTTCAGTCTAAAATGGATGAGAAGAATGCTCATATCCCCTCAGAAAGTATTGCTTGTAAGA
GGGACCCAGAACAGGCAAAAGAAGAGCCAGGGCATATTCCCACACAGCATGTGGAGGAGGCTGTGAATGAG
GTAGACAACGAAAACACCCAGCAGAAAGATGATGAGAGTGATGCCCCATGCAGCTCTCTTGGGTTGTCGAGT
AGTGGAAGTGGTGATGCTGCTAGGGCACCAAAATCGGTGCCAAGGCCTAAAAGATTGACCTCTTCAACCTAC
AACCTAAGACACGCTCATTCTCTGGGCTCCTTGGATGCTTCAAAAGTGACTTCAGAAAAGGAAGCTGCACAAG
TAAACCCCATAATGCCAAAGGAAAATGGAGCTTCAGAGAGTGGAGACCCCCTAGATGAGGACGATGTTGACA
CCGTGGTAGATGAACAGCCAAAGTTTATGGAATGGTGTGCTGAGGAGGAGAACCAAGAGCTCATCGCCAACT
TCAATGCCCAGTACATGAAAGTTCAGAAGGGCTGGATCCAGTTGGAGAAAGAAGGACAGCCAACACCAAGA
GCAAGGAACAAATCAGATAAACTGAAAGAGATTTGGAAAAGCAAGAAAAGGTCACGGAAATGTAGGAGTTC
ATTGGAGAGTCAGAAGTGTTCTCCTGTTCAGATGCTCTTTATGACAAACTTTAAATTATCTAATGTTTGTAAATG
GTTCTTAGAGACAACTGAAACCCGGTCTCTAGTCATTGTGAAGAAGCTCAATACTCGCCTTCCAGGAGACGTT
CCCCCTGTCAAGCATCCTCTTCAGAAATACGCTCCTTCCAGCCTATATCCCAGTTCACTACAGGCTGAGCGCTTG
AAAAAGCACTTGAAGAAATTTCCTGGAGCTACCCCTGCTAAGAATAATTGGAAAATGCAGAAGCTCTGGGCCA
AATTTCGAGAGAATCCTGATCAAGTGGAGCCAGAAGATGGCAGTGATGTCAGCCCCGGCCCTAATTCTGAAG
ACAGCATAGAGGAAGTCAAGGAAGATAGAAACAGTCATCCTCCAGCAAACCTGCCCACTCCAGCCAGTACCC
GGATTCTTAGAAAATATTCCAATATTCGAGGAAAGCTCAGAGCCCAGCAACGTTTAATCAAGAATGAGAAAAT
GGAATGCCCAGATGCTCTGGCTGTGGAAAGTAAGCCAAGTCGTAAGAGCGTATGCATCAACCCTCTGATGTCC
CCCAAGCTTGCCCTGCAAGTGGATGCAGATGGGTTTCCTGTTAAGCCCAAGAGTACTGAAGGAATGAAGGGA
AGGAAGGGGAAGCAGGTGTCTGAAATCTTGCCTAAAGCAGAAGTTCAGAGTAAACGCAAGAGAACAGAAGG
CAGCAGCCCTCCAGATAGTAAGAACAAGGGGCCTACGGTGAAAGCCAGCAAAGAAAAGCATGCTGATGGAG
CCACCAAAACCCCTGCTGCCAAGAGGCCAGCTGCAAGGGACAGAAGCAGCCAACCCCCCAAAAAGACGTCTT
TGAAAGAGAATAAAGTGAAGATCCCTAAAAAGTCCGCTGGGAAGAGCTGCCCTCCCTCCAGGAAAGAAAAAG
AGAATACAAACAAAAGGCCTTCCCAGTCTATTGCCTCGGAAACACTGACGAAACCTGCAAAACAGAAGGGGG
CCGGTGAATCCTCTTCAAGGCCTCAGAAAGCCACGAATAGGAAGCAGAGTAGTGGAAAGACTCGGGCCAGAC
CCTCAACGAAAACCCCAGAGAGCAGTGCAGCTCAGAGAAAGCGAAAGCTGAAGGCAAAGCTGGACTGTTCG
CACAGCAAACGGAGGCGGCTGGATGCAAAG
