# Supplementary material for: PALI1 facilitates DNA and nucleosome binding by PRC2 and triggers an allosteric activation of catalysis
Source: Nat Commun. 2021 Jul 28;12:4592. doi: 10.1038/s41467-021-24866-3 (PMC8319299; doi:10.1038/s41467-021-24866-3)
Supplement: Supplementary file 4 — Description of additional supplementary files [file 41467_2021_24866_MOESM4_ESM.docx]

Description of additional supplementary information

Title: Supplementary Dataset

Description: *The summary of the PRC2 methylome in vivo and in vitro. Residues with a position probability of less than 0.95 were indicated with red text and probability scores shown in parentheses. Residues from peptides that were ambiguous between EZH1 and EZH2 are indicated by an asterisk.*"
