# Supplementary material for: PALI1 facilitates DNA and nucleosome binding by PRC2 and triggers an allosteric activation of catalysis
Source: Nat Commun. 2021 Jul 28;12:4592. doi: 10.1038/s41467-021-24866-3 (PMC8319299; doi:10.1038/s41467-021-24866-3)
Supplement: Supplementary file 5 — Reporting Summary [file 41467_2021_24866_MOESM5_ESM.pdf]

nature research

Corresponding author(s): Chen Davidovich

Last updated by author(s): Jun 23, 2021

Reporting Summary

Nature Research wishes to improve the reproducibility of the work that we publish. This form provides structure for consistency and transparency in reporting. For further information on Nature Research policies, see Authors & Referees and the Editorial Policy Checklist.

Statistics

For all statistical analyses, confirm that the following items are present in the figure legend, table legend, main text, or Methods section.

n/a	Confirmed	
☐	☒	The exact sample size (n) for each experimental group/condition, given as a discrete number and unit of measurement
☐	☒	A statement on whether measurements were taken from distinct samples or whether the same sample was measured repeatedly
☒	☐	The statistical test(s) used AND whether they are one- or two-sided<br>*Only common tests should be described solely by name; describe more complex techniques in the Methods section.*
☒	☐	A description of all covariates tested
☒	☐	A description of any assumptions or corrections, such as tests of normality and adjustment for multiple comparisons
☐	☒	A full description of the statistical parameters including central tendency (e.g. means) or other basic estimates (e.g. regression coefficient) AND variation (e.g. standard deviation) or associated estimates of uncertainty (e.g. confidence intervals)
☐	☒	For null hypothesis testing, the test statistic (e.g. F, t, r) with confidence intervals, effect sizes, degrees of freedom and P value noted<br>*Give P values as exact values whenever suitable.*
☒	☐	For Bayesian analysis, information on the choice of priors and Markov chain Monte Carlo settings
☒	☐	For hierarchical and complex designs, identification of the appropriate level for tests and full reporting of outcomes
☒	☐	Estimates of effect sizes (e.g. Cohen's d, Pearson's r), indicating how they were calculated

Our web collection on statistics for biologists contains articles on many of the points above.

Software and code

Policy information about availability of computer code

Data collection	Amersham Typhoon Scanner Control software 1.1, PHERAstar 4.00R4, ChemiDoc™ imager, BD LSRFortressa™ X-20 analyser, BD Influx™ cell sorter
Data analysis	PyMoL 2.0, ImageJ 1.51j8, GraphPad Prism 8, MaxQuant 1.6.2.0, CCP4 7.0.042, PHASER 2.7.17, REFMAC5 5.8.0158, PHENIX1.17.1, COOT 0.8.8, BD FACSDiva™, FlowJo, Matlab 9.6.0.1072779

For manuscripts utilizing custom algorithms or software that are central to the research but not yet described in published literature, software must be made available to editors/reviewers. We strongly encourage code deposition in a community repository (e.g. GitHub). See the Nature Research guidelines for submitting code & software for further information.

Data

Policy information about availability of data

All manuscripts must include a data availability statement. This statement should provide the following information, where applicable:

- Accession codes, unique identifiers, or web links for publicly available datasets
- A list of figures that have associated raw data
- A description of any restrictions on data availability

Coordinates and structure factors have been deposited in the Protein Data Bank under accession codes 6V3X (http://doi.org/10.2210/pdb6V3X/pdb) and 6V3Y (http://doi.org/10.2210/pdb6V3Y/pdb). The mass spectrometry data have been deposited to Monash University research repository Figshare, with DOI https://doi.org/10.26180/14752509.

Field-specific reporting

Please select the one below that is the best fit for your research. If you are not sure, read the appropriate sections before making your selection.

☒ Life sciences ☐ Behavioural & social sciences ☐ Ecological, evolutionary & environmental sciences

For a reference copy of the document with all sections, see nature.com/documents/nr-reporting-summary-flat.pdf

Life sciences study design

All studies must disclose on these points even when the disclosure is negative.

Sample size	All experiments were carried out in at least three replicates to ensure independent experiments are reproducible. At least three replicates were performed, since this number is feasible and allows statistical analysis.
Data exclusions	No data were excluded from the analyses.
Replication	All experimental findings were reproducible.
Randomization	Randomization was not applicable, not practical and did not apply to the type of biochemical assays carried out here.
Blinding	Blinding was not applicable, not practical and did not apply to the type of biochemical assays carried out here.

Reporting for specific materials, systems and methods

We require information from authors about some types of materials, experimental systems and methods used in many studies. Here, indicate whether each material, system or method listed is relevant to your study. If you are not sure if a list item applies to your research, read the appropriate section before selecting a response.

Materials & experimental systems

n/a	Involved in the study	
☐	☒	Antibodies
☐	☒	Eukaryotic cell lines
☒	☐	Palaeontology
☒	☐	Animals and other organisms
☒	☐	Human research participants
☒	☐	Clinical data

Methods

n/a	Involved in the study	
☒	☐	ChIP-seq
☐	☒	Flow cytometry
☒	☐	MRI-based neuroimaging

Antibodies

Antibodies used	anti-Flag, anti-Flag HRP-conjugated, anti-LCOR, anti-actin, anti-EZH2, anti-H3, anti-H3K27me3, anti-EED, anti-CD235a, anti-CD44, anti-mouse HRP-conjugated
Validation	We validated antibodies for the detection of EZH2 (Active Motif #39875) and EED (Abclonal #A12773) using knockout. We validated the Anti-LCOR antibody using the overexpression of PALI1 in cells, and the same antibody has been validated by others using knockout (Conway et al. Mol Cell. 2018; DOI: 10.1016/j.molcel.2018.03.005). Antibodies for the detection of H3K27me3 (Merck #07-449), Flag (Sigma #A8592), and Actin (Sigma #A2066) are commonly used (>100 citations each) and were validated by the manufacturer. Antibody for the detection of CD44 (Biolegend #103011, Biolegend #103007) using flow cytometry was used for this application in previous studies (e.g. Biton et al. Cell 2018; DOI: 10.1016/j.cell.2018.10.008). Antibody for the detection of CD235a (BioLegend #349108) using flow cytometry was used for this application in previous studies (e.g. Krampitz et al. PNAS 2016; DOI: 10.1073/pnas.1600007113).

Eukaryotic cell lines

Policy information about cell lines

Cell line source(s)	K562 cells were purchased from ATCC by our lab. HeLa cells were purchased from ATCC and were a gift from the Michael Lazarou Lab, Monash University. HEK293T cells were a gift from Jose Polo lab, Monash University, with the original commercial source is unknown.
Authentication	HeLa cells were authenticated by the Johns Hopkins GRCF Fragment Analysis Facility using STR profiling (Lazarou et al. Nature 2015; doi: 10.1038/nature14893). K562 and HEK293T cells were not authenticated.

Mycoplasma contamination	All cell lines are routinely tested for mycoplasma contamination using PCR. The cell lines used in this study were tested negative.
Commonly misidentified lines (See ICLAC register)	No commonly misidentified cell lines were used in this study.

Flow Cytometry

Plots

Confirm that:

☒ The axis labels state the marker and fluorochrome used (e.g. CD4-FITC).

☒ The axis scales are clearly visible. Include numbers along axes only for bottom left plot of group (a 'group' is an analysis of identical markers).

☒ All plots are contour plots with outliers or pseudocolor plots.

☒ A numerical value for number of cells or percentage (with statistics) is provided.

Methodology

Sample preparation	K562 cells were centrifuged at 500 g for 5 minutes, and the supernatant removed. For staining, cells were resuspended at a density of 2x10^7 cells/mL and incubated for 15 minutes on ice in flow cytometry buffer with the appropriate antibody concentration. The cells were centrifuged again at 500 g for 5 minutes and the supernatant removed, then washed with antibody-free flow cytometry buffer. The cells were then resuspended in flow cytometry buffer (PBS supplemented with 10 % FBS and 615 µM EDTA) to a density of approximately 10^7 cells/mL, ran through a cell strainer (Falcon 352235) and kept on ice.
Instrument	BD LSRFortressa™ X-20 analyser, BD Influx™ cell sorter
Software	Data were analysed using BD FACSDiva™ and GraphPad Prism.
Cell population abundance	For all experiments that involved cell sorting, the sorted cells were subjected to either analytical FACS or immunoblotting 7 days later. The resulting data showed that the cells had been sorted to a sufficient purity.
Gating strategy	The first preliminary gate was set with SSC-A/FSC-A on the axes, and the intact cells were defined to primarily include the main clustered population of these events. The second preliminary gate was set with FSC-A/FSC-H on the axes, and single cells defined to be those falling directly along the diagonal of that graph. The gate for GFP and dTomato positive cells was set to exclude the cells within an untransduced sample. A graphical representation of gating strategies used in this study is included in the Supplementary Notes.

☒ Tick this box to confirm that a figure exemplifying the gating strategy is provided in the Supplementary Information.
